# Molecular Modulation of Human α7 Nicotinic Receptor by Amyloid-β Peptides

**DOI:** 10.3389/fncel.2019.00037

**Published:** 2019-02-08

**Authors:** Matías Lasala, Camila Fabiani, Jeremías Corradi, Silvia Antollini, Cecilia Bouzat

**Affiliations:** Instituto de Investigaciones Bioquímicas de Bahía Blanca (INIBIBB), Departamento de Biología, Bioquímica y Farmacia, Universidad Nacional del Sur-Consejo Nacional de Investigaciones Científicas y Técnicas (CONICET), Bahía Blanca, Argentina

**Keywords:** nicotinic receptor, patch-clamp recordings, single-channel currents, Cys-loop receptor, amyloid peptide, crystal violet

## Abstract

Amyloid β peptide (Aβ) is a key player in the development of Alzheimer’s disease (AD). It is the primary component of senile plaques in AD patients and is also found in soluble forms. Cholinergic activity mediated by α7 nicotinic receptors has been shown to be affected by Aβ soluble forms. To shed light into the molecular mechanism of this effect, we explored the direct actions of oligomeric Aβ_1–40_ and Aβ_1–42_ on human α7 by fluorescence spectroscopy and single-channel recordings. Fluorescence measurements using the conformational sensitive probe crystal violet (CrV) revealed that in the presence of Aβ α7 undergoes concentration-dependent conformational changes. Exposure of α7 to 100 pM Aβ changes CrV K_D_ towards that of the desensitized state. However, α7 is still reactive to high carbamylcholine (Carb) concentrations. These observations are compatible with the induction of active/desensitized states as well as of a novel conformational state in the presence of both Aβ and Carb. At 100 nM Aβ, α7 adopts a resting-state-like structure which does not respond to Carb, suggesting stabilization of α7 in a blocked state. In real time, we found that Aβ is capable of eliciting α7 channel activity either in the absence or presence of the positive allosteric modulator (PAM) PNU-120596. Activation by Aβ is favored at picomolar or low nanomolar concentrations and is not detected at micromolar concentrations. At high Aβ concentrations, the mean duration of activation episodes elicited by ACh in the presence of PNU-120596 is significantly reduced, an effect compatible with slow open-channel block. We conclude that Aβ directly affects α7 function by acting as an agonist and a negative modulator. Whereas the capability of low concentrations of Aβ to activate α7 could be beneficial, the reduced α7 activity in the presence of higher Aβ concentrations or its long exposure may contribute to the cholinergic signaling deficit and may be involved in the initiation and development of AD.

## Introduction

Alzheimer’s disease (AD) is a progressive neurodegenerative disease characterized by loss of memory, multiple cognitive impairments and changes in personality and behavior. Memory impairment in AD is associated with neuronal degeneration as well as synaptic damage. Although AD is a multifactorial disease, accumulation of amyloid-β peptides (Aβ) is one of the major pathological factors. Accumulation phase starts with low molecular weight fractions of Aβ (monomers, dimers, or trimers) and continues with larger oligomers or insoluble amyloid fibrils (Sadigh-Eteghad et al., 2014). Although plaques remain the principal identifiers and predictors of Alzheimer’s disease, a clear paradigm shift has occurred that emphasizes the primacy of Aβ oligomers in disease causation (Lacor et al., 2004; Shankar et al., 2008; Hayden and Teplow, 2013; Collins-Praino et al., 2014). Despite all research efforts, there are still many unsolved aspects regarding the molecular mechanisms underlying Aβ pathogenic actions. One of these mechanisms involves the interaction of Aβ with synaptic receptors, which consequently emerge as novel druggable sites to restore cognitive functions in AD patients (Kandimalla and Reddy, 2017).

Cholinergic neurons with a pivotal role in learning and memory are mainly involved in the pathogenesis of AD. Indeed, inhibitors of acetylcholinesterase (AChE), which by decreasing ACh breakdown enhance cholinergic neurotransmission, are to date one of the main specific therapeutic drugs, although their efficacy is limited (Kandimalla and Reddy, 2017).

The α7 nicotinic acetylcholine receptor (nAChR) has been shown to be associated with AD. α7 is highly expressed in hippocampus, cortex and several subcortical limbic regions and is involved in cognition, sensory processing information, attention, working memory, and reward pathways (Lendvai et al., 2013). Reduction of α7 in brain, particularly in the hippocampus, has been reported in AD patients (Buckingham et al., 2009; Dineley et al., 2015).

α7 is a pentameric ligand-gated ion channel that responds to ACh by opening an intrinsic channel permeable to cations that triggers rapid membrane depolarization and calcium influx (Wonnacott, 2014). However, α7 also acts as a metabotropic receptor and triggers several signal transduction pathways as well as the release of calcium from intracellular stores (Kabbani et al., 2013; Egea et al., 2015; Guan et al., 2015; Corradi and Bouzat, 2016; Bouzat et al., 2018). This metabotropic activity has been associated to synaptic plasticity and neuroprotection, including against Aβ damage (Buckingham et al., 2009; Inestrosa et al., 2013; Jin et al., 2015).

Enhancement of α7 activity is emerging as a therapeutic strategy for cognitive impairment in AD. Positive allosteric modulators (PAMs) are the most promising therapeutic compounds because they maintain the temporal and spatial characteristics of endogenous activation, are more selective than agonists, and reduce tolerance due to desensitization (Chatzidaki and Millar, 2015; Terry et al., 2015; Corradi and Bouzat, 2016; Echeverria et al., 2016; Yang et al., 2017; Bouzat et al., 2018). Based on their effects on macroscopic currents, PAMs have been classified as type I PAMs, that mainly enhance agonist-induced peak currents, and type II PAMs, that enhance agonist-elicited currents and also decrease desensitization and recover receptors from desensitized states (Bertrand and Gopalakrishnan, 2007; Grønlien et al., 2007; Andersen et al., 2016).

Both α7 agonist- and antagonist-like actions of Aβ have been described in different cells and tissues (Wu et al., 2004; Khan et al., 2010; Li et al., 2011; Parri et al., 2011; Sadigh-Eteghad et al., 2014; Liu et al., 2015; Yan et al., 2015). Studies have been focused mainly on evaluation of the effects of Aβ on α7 metabotropic activity, which includes signaling pathways, such as ERK/MAPK, PI3K/AKT, JAK-2/STAT-3 and intracellular calcium mobilization. The acute effects of Aβ on α7 electrical activity have been explored at the macroscopic current level. Despite problems of comparability and some dissimilar results, probably due to variations on the aggregation state and concentration of amyloid peptides (see Buckingham et al., 2009), the consensus indicates that low concentrations of Aβ (picomolar) activate α7 whereas higher concentrations lead to current inhibition (Liu et al., 2001; Dineley et al., 2002; Grassi et al., 2003; Pym et al., 2005; Parri et al., 2011).

To shed light into the molecular mechanism of the direct action of Aβ on α7, we expressed human α7 on mammalian cells and evaluated functional effects by fluorescence spectroscopic measurements and electrophysiological recordings. We used Aβ preparations enriched in oligomeric forms since these species were shown to be involved in cognitive impairment, inhibition of long-term potentiation, memory loss and α7 modulation (Walsh et al., 2002; Wang et al., 2002; Cleary et al., 2005; Parri et al., 2011). By taking advantage of the potential of single-channel recordings in providing information unattainable by macroscopic measurements, we deciphered the direct molecular effects of oligomeric Aβ as activator and inhibitor of α7 channels. By using a fluorescent conformational probe, we revealed that Aβ elicits different concentration-dependent conformational changes and induces novel conformational states.

## Materials and Methods

### Drugs

Acetylcholine (ACh) and human amyloid-β1–42 (Aβ_1–42_) were purchased from Sigma-Aldrich (St. Louis, MO, USA); NS-1738 N-(5-Chloro-2-hydroxyphenyl)-N′-[2-chloro-5-(trifluoromethyl) phenyl]urea, PNU-120596 [N-(5-Chloro-2,4-dimethoxyphenyl)-N′-(5-methyl-3-isoxazolyl)-urea] and rat amyloid-β1–40 (Aβ_1–40_) were obtained from Tocris Biosciences (Bristol, UK).

### Receptor Expression

BOSC-23 cells, derived from HEK 293 cells (Pear et al., 1993), were transfected by calcium phosphate procedure with human α7 cDNA subcloned in pRBG4 vector (Bouzat et al., 1994). Plasmids harboring cDNAs of the α7 chaperone proteins Ric-3 and NACHO were incorporated to favor α7 expression (Bouzat et al., 2008; Andersen et al., 2016; Nielsen et al., 2018). All transfections were carried out for about 12–18 h in Dulbecco’s Modified Eagle Medium (DMEM) with 10% fetal bovine serum and terminated by exchanging the medium. Cells were used for single-channel recordings 2–4 days after transfection. To facilitate identification of transfected cells, a separate plasmid encoding green fluorescent protein was included in all transfections.

### Amyloid-β Peptide Preparations

Aβ_1–40_ or Aβ_1–42_ were resuspended in dimethyl sulfoxide at a concentration of 10 mg/ml (peptide stock solution) and stored in aliquots at −80°C. Aβ oligomers were prepared immediately prior to use according to previously published methods (Uranga et al., 2016; Pascual et al., 2017). Briefly, aliquots of peptide stock (10 μl) were added to 280 μl of phosphate buffered saline (PBS; pH 7.4) and stirred continuously (300 rpm) for 120 min and stored at 4°C until use.

Transmission electron microscopy (TEM) studies were carried out according to previously published methods with slight modifications (Uranga et al., 2016; Pascual et al., 2017). Briefly, 10 μl media containing the peptide was placed on a carbon-coated grid and incubated for 60 s. Ten microliters of 0.5% glutaraldehyde was added to the grid followed by incubation for an additional 60 s. The grid was then washed with drops of water and dried. Finally, the grid was stained for 2 min with 2% uranyl acetate and air dried. The grid was subsequently examined under a Jeol 100 Cx II electron microscope (Uranga et al., 2016). TEM photomicrographs showed that Aβ preparations included spheroidal structures individually or in small groups (Supplementary Figure S1). These ultrastructural forms were compatible with a heterogeneous array of oligomers, thus discarding the presence of fibrils (Uranga et al., 2016; Pascual et al., 2017).

### Single-Channel Recordings

Single-channel recordings were obtained in the cell-attached patch configuration. The bath and pipette solutions contained 142 mM KCl, 5.4 mM NaCl, 1.8 mM CaCl_2_, 1.7 mM MgCl_2_ and 10 mM HEPES (pH 7.4). For potentiation, 1 μM PNU-120596 or 10 μM NS-1738 were added to the pipette solution together with ACh.

Single-channel currents were digitized at 5–10 μs intervals, low-pass filtered at a cut-off frequency of 10 kHz using an Axopatch 200B patch-clamp amplifier (Molecular Devices Corp., CA, USA). Single-channel events were idealized by the half amplitude threshold criterion using the program QuB 2.0.0.28 (Qin et al., 1996, 1997; State University of New York at Buffalo) with a digital low-pass filter at 9 kHz. A filter of 3 kHz was used in recordings with PNU-120596 to facilitate the analysis. The open and closed time histograms obtained from idealization were fitted by the maximum interval likelihood (MIL) function in QuB (Qin et al., 1996, 1997), with a dead time of 0.1 ms. This analysis was performed by sequentially adding an open and/or closed state to a starting C ↔ O model in order to properly fit the corresponding histograms (Fabiani et al., 2018; Lasala et al., 2018). Final models contained five-six closed states and three-four open states for α7 in the presence of ACh plus PNU-120596, five-six closed states and three open states for α7 in the presence of ACh plus NS-1738, or three closed states and one-two open states for α7 in the presence of ACh and absence of PAMs.

Clusters were identified as a series of closely separated openings preceded and followed by closings longer than a critical duration. Different critical closed times were calculated by MIL between each closed component. Critical times between the third and fourth closed components for α7 in the presence of PNU-120596 (~30–60 ms) or NS-1738 (~2–8 ms) were selected in QuB to chop the idealized data and create a sub-data set that only contained clusters to define mean cluster duration.

### Fluorimetric Measurements

Fluorimetric measurements were performed in a SLM model 4800 fluorimeter (SLM Instruments, Urbana, IL, USA) using a vertically polarized light beam from an Hannovia 200-W mercury/xenon arc obtained with a Glan-Thompson polarizer (4 nm excitation and emission slits).

Crystal violet (CrV) was used as a probe to detect conformational changes (Lurtz and Pedersen, 1999; Sun et al., 2017; Fabiani et al., 2018). nAChR-rich membranes were prepared from *Torpedo californica* electric tissue as described previously (Fernández Nievas et al., 2008; Perillo et al., 2012; Fabiani et al., 2018). CrV experiments using *Torpedo* membranes or α7-expressing cells were conducted as described previously for the *Torpedo* nAChR (Fernández Nievas et al., 2008; Perillo et al., 2012; Fabiani et al., 2018). *Torpedo* membranes or BOSC-23 cells expressing α7 were resuspended on phosphate saline buffer with a protein concentration of 100 μg/ml or up to an absorbance value of ~0.5 at 280 nM measured on a Jasco V-630 spectrophotometer (JASCO Deutschland GmbH), respectively. The suspended cells or membranes were incubated with Aβ oligomers for 20 min. For measurements conducted in the desensitized state, the membrane or cell suspensions were additionally incubated for 15 min with 1 mM or 20 mM carbamylcholine (Carb), respectively. The suspensions were subsequently titrated with increasing concentrations of CrV (in water). After each addition of CrV, the samples were incubated for 15 min before obtaining the fluorescence emission spectra. CrV was excited at 600 nm, and the fluorescence emission spectra were collected from 605 to 700 nm. Before the first addition of CrV, a background fluorescence emission spectrum was obtained for each sample. The spectrum was then subtracted from the emission spectra obtained in the presence of CrV and the maximum intensity (at 623–625 nm) was measured. To determine the CrV dissociation constant (K_D_), the value of the CrV maximum fluorescence emission was plotted as a function of the logarithmic CrV concentration (M). The resulting sigmoid curve was fitted by the Boltzmann function and the K_D_ was calculated using the program Origin 7.0 (OriginLab Corporation).

### Statistical Analysis

Intergroup comparisons were carried out using one-way analyses of variance (ANOVA), Dunnett’s multiple comparisons test (Graphpad PRISM software). The values represent the average ± SD of the total number of samples indicated. Statistically significance difference was established at *p*-values <0.05.

## Results

### Amyloid β Oligomers Induce Conformational Changes in α7

To examine the potential of oligomeric Aβ peptides to directly affect α7, we first used the conformational-sensitive probe CrV and measured its affinity for the receptor before and after exposure to Aβ by fluorescence spectroscopy.

**Figure 1:**
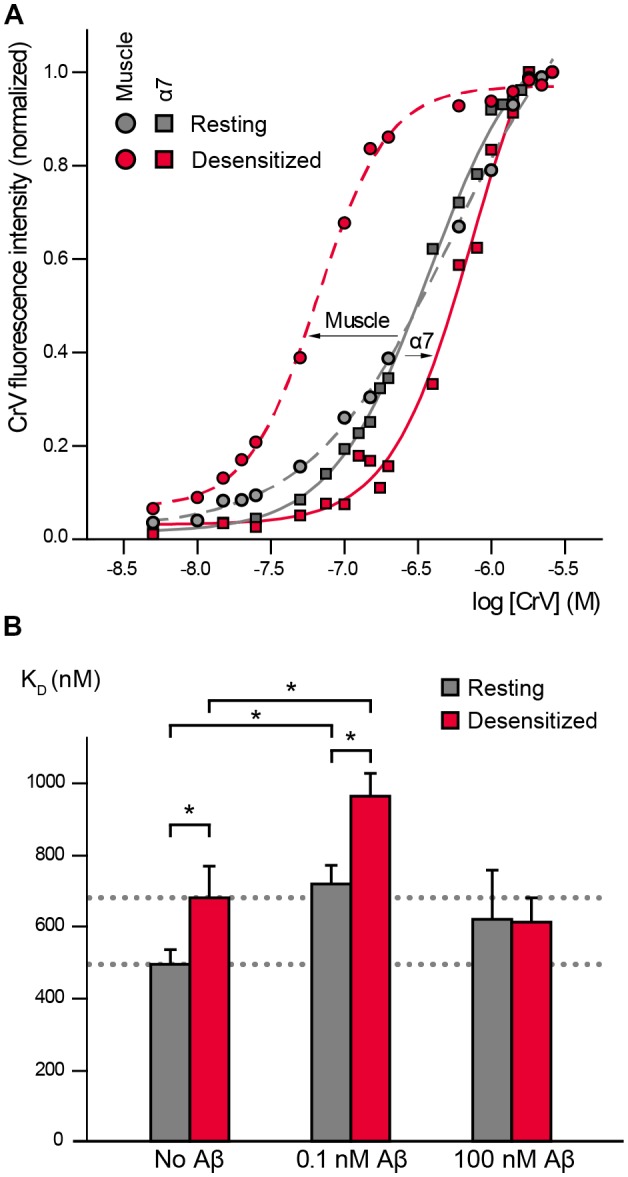
α7 conformational changes depend on Amyloid β peptide (Aβ) concentrations. **(A)** Crystal violet (CrV) titration curves obtained with muscle nicotinic acetylcholine receptor (nAChR) from *T. californica* rich membranes (circles) and with human α7 (squares) in the absence (gray, Resting) and presence of carbamylcholine (Carb; red, desensitized). The arrows indicate the changes from resting to desensitized conditions for each nAChR type. **(B)** Changes in K_D_ values of CrV by the exposure to low (0.1 nM) and high (100 nM) concentrations of Aβ_1–40_. K_D_ values of CrV were calculated from the titration curves, in the absence and presence of 20 mM Carb (gray and red columns, respectively). Each column corresponds to the average ± SD of more than four independent experiments. Statistically significant differences are shown by asterisks, *p < 0.05.

CrV has been extensively used for studies in the *Torpedo* muscle nAChR where it shows low affinity for the resting state (K_D_ values ~400 nM, Fernández Nievas et al., 2008; Perillo et al., 2012) and high affinity for the desensitized state (K_D_ values ~60 nM; Fernández Nievas et al., 2008; Perillo et al., 2012; Fabiani et al., 2018; Figure 1A).

We first determined if CrV can be used as a conformational probe for the human α7 receptor. Suspensions of BOSC-23 cells expressing α7 were titrated with increasing concentrations of CrV. Saturable CrV binding yielded a K_D_ value of 492 ± 41 nM for resting α7 receptors, indicating an affinity similar to that for the muscle nAChR in the resting state. Interestingly, measurements in the desensitized state, which was induced by 20-min preincubation with Carb, yielded a K_D_ value of 680 ± 89 nM for CrV binding (Figures 1A,B). The maximal fluorescence intensities of the samples were similar at resting and desensitized conditions, thus discarding that the lower affinity for the desensitized state corresponds to reduced binding of the probe in the latter state. These results indicate that CrV saturates its binding sites at both conditions with different affinities. Thus, opposite to its binding affinity profile for the muscle nAChR, CrV shows higher affinity for resting than for desensitized states in α7.

We next determined the effects of low (0.1 nM) and high (100 nM) Aβ_1–40_ concentrations on α7 conformation by measuring CrV K_D_ values to resting and desensitized states (Figure 1B).

Exposure of α7 in the resting state (in the absence of Carb) to 0.1 nM Aβ_1–40_ increased the CrV K_D_ towards that of the desensitized state, indicating that amyloid peptides induce conformational changes in α7. Interestingly, subsequent addition of Carb induced a further displacement to higher K_D_ values, suggesting that the agonist can lead to further conformational changes (Figure 1B). K_D_ values for desensitized conditions were statistically significantly different in the absence and presence of Aβ (Figure 1B). Similar results were obtained with 0.1 nM Aβ_1–42_.

On the other hand, incubation of resting α7 receptors with 1,000-fold higher Aβ_1–40_ concentration (100 nM) did not induce statistically significant changes in the CrV K_D_ value (Figure 1B). Furthermore, the K_D_ value remained constant even after addition of 20 mM Carb, indicating that α7 was not reactive to the agonist. Similar results were obtained with high (100 nM) Aβ_1–42_.

### Oligomeric Amyloid-β Peptides at Low Concentrations Trigger Human α7 Channel Opening

Once established that oligomeric Aβ peptides induce conformational changes that are sensitive to concentration, we took advantage of the potential of single-channel recordings to reveal the mechanistic basis of this modulation.

**Figure 2:**
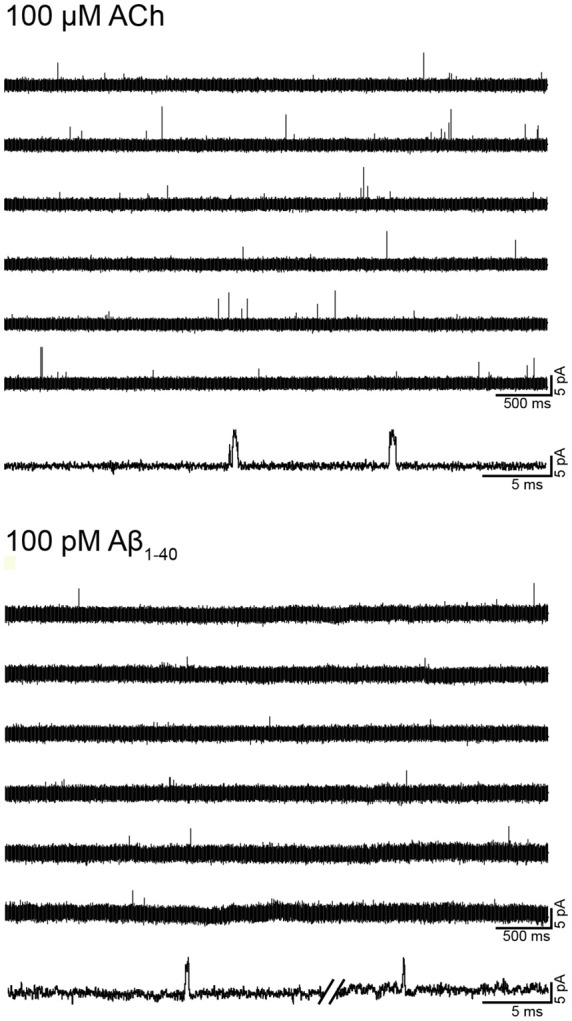
Single-channel activity elicited by Aβ. Single-channel recordings were performed in the cell-attached patch configuration at −70 mV membrane potential. Representative α7 single-channel traces from a continuous recording in the presence of 100 μM acetylcholine (ACh) or 100 pM Aβ_1–40_. For each condition, a trace at higher temporal resolution is shown (bottom traces). Channel openings are shown as upward deflections. Filter: 9 kHz.

To first determine if Aβ can activate human α7, we examined single-channel activity from BOSC-23 cells expressing the receptor (Figure 2). In the presence of 100 μM ACh, α7 exhibits single brief openings flanked by long closings, or less often, several openings in quick succession, which are called bursts (Bouzat et al., 2008; Andersen et al., 2016). The mean open duration was 0.36 ± 0.07 ms (*n* = 3) and the mean burst duration was 0.77 ± 0.21 ms** (***n* = 3; Figure 2). No α7 channel activity was detected in the absence of agonist. However, 100 pM Aβ_1–40_ elicited the typical α7 channel openings (Figure 2). The number of active patches was lower than in the presence of ACh (28% transfected cells showed channel activity). Channel activity was not detected if Aβ_1–40_ was increased to 10 nM and 100 nM (*n* = 16 patches of different cells and transfections), indicating that activation is favored at low concentrations. These experiments were performed in parallel with recordings with ACh as the agonist to discard that the lack of channel activity was due to the lack of functional expression.

**Figure 3:**
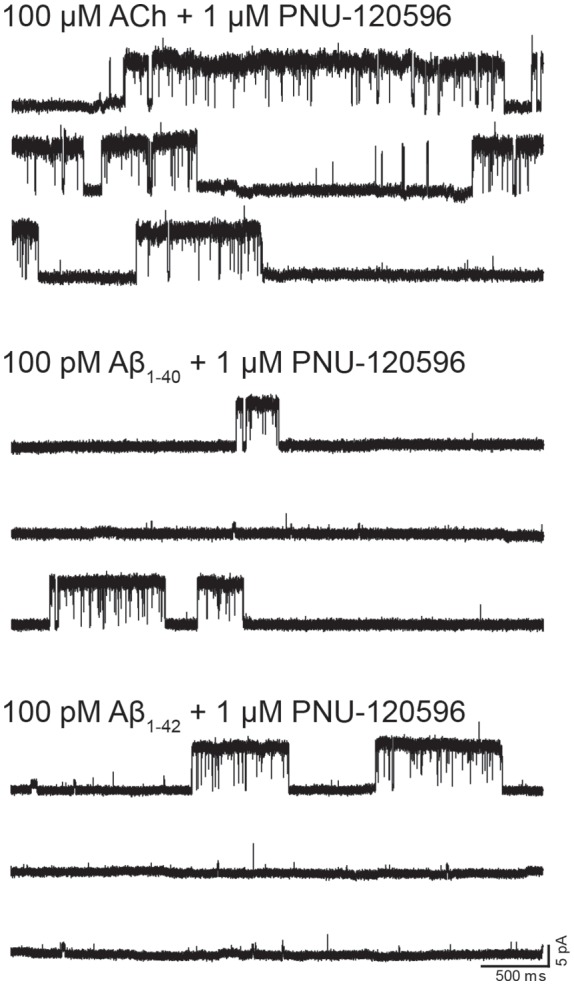
Single-channel activity elicited by Aβ in the presence of N-(5-Chloro-2,4-dimethoxyphenyl)-N′-(5-methyl-3-isoxazolyl)-urea (PNU-120596). Single-channel recordings were performed in the cell-attached patch configuration at −70 mV membrane potential. Representative α7 single-channel traces from a continuous recording in the presence of the type II positive allosteric modulator (PAM) PNU-120596 (1 μM) together with 100 μM ACh, 100 pM Aβ_1–40_ or 100 pM Aβ_1–42_. Channel openings are shown as upward deflections.

We next performed recordings in the presence of the type II PAM PNU-120596, which increases the probability of agonist-elicited channel opening and, consequently, favors the detection of infrequent opening events (daCosta et al., 2011; Andersen et al., 2016). By itself, PNU-120596 cannot elicit channel activation (Hurst et al., 2005). In the presence of 1 μM PNU-120596, α7 channel activity elicited by ACh (100 μM) appears in long activation periods of high frequency, named clusters, whose mean duration is about 1–3 s (Figure 3, Table 1; Andersen et al., 2016).

**Table 1:** Activation of α7 by Amyloid β peptide (Aβ) in the presence of N-(5-Chloro-2,4-dimethoxyphenyl)-N′-(5-methyl-3-isoxazolyl)-urea (PNU)-120596.

[ACh] (μM)	Aβ	[Aβ] (nM)	% active patches (n)	Mean cluster duration (ms)
100	No	0	100 (6)	2263 ± 990
0	Aβ_1–40_	0.1	42.9 (7)	762 ± 316
0		1	22.2 (18)	2016 ± 1364
0		10	30.0 (10)	5229 ± 1392*
0		100	0 (9)	Nd
0	Aβ_1–42_	0.1	77.8 (9)	1401 ± 805
0		1	46.2 (13)	1208 ± 946
0		10	33.3 (9)	1751 ± 766
0		100	0 (6)	Nd

Single-channel currents from cells expressing human α7 were recorded in the presence of 1 μM PNU-120596 plus 100 μM acetylcholine (ACh) or Aβ at the indicated concentrations. The mean cluster duration was obtained from the corresponding histograms. Nd: not detected. Statistical comparisons were performed against the corresponding control condition using analyses of variance (ANOVA; Dunnett’s multiple comparisons test). *p < 0.05.

In the absence of ACh, Aβ_1–40_ or Aβ_1–42_ at a low concentration (100 pM) elicited clusters of PNU-120596-potentiated α7 channels (Figure 3). Again, the proportion of active patches was lower than in the presence of ACh. Whereas almost all patches (>90%) showed channel activity with ACh, the percentage of active patches was reduced in the presence of Aβ_1–40_ or Aβ_1–42_ as agonists (Table 1). Moreover, as Aβ concentration increased, the number of patches showing α7 activity decreased. As shown in Table 1, at 100 pM 43% and 78% of the patches showed clusters elicited by Aβ_1–40_ or Aβ_1–42_, respectively, but no channel activity was detected at 100 nM. Thus, also in the presence of a PAM, activation by Aβ is favored at picomolar or low nanomolar concentrations.

Although the frequency of clusters is usually variable among patches from different cell transfections, it was systematically lower in the presence of Aβ with respect to ACh as illustrated in typical recordings shown in Figure 3. For a better comparison, recordings with ACh or Aβ were performed in the same batch of transfected cells.

In the presence of ACh and PNU-120596, each cluster is composed of two or three bursts that contain long-duration openings separated by brief closings (daCosta et al., 2011; Andersen et al., 2016). Clusters elicited by Aβ in the presence of PNU-120596 showed the typical architecture observed with ACh (Figure 3). The analysis showed no statistically significant differences in the mean cluster duration at all tested concentrations, except for 10 nM Aβ_1–40_ at which clusters were slightly prolonged (Table 1).

The results confirm that Aβ at picomolar and low nanomolar concentrations can trigger activation of α7 channels.

### Amyloid-β Peptides Decrease the Duration of α7 Activation Episodes

We next explored the effect of Aβ on ACh-elicited channels. To this end, we recorded channels activated by 100 μM ACh in the presence of Aβ_1–42_ (10 and 100 nM). The mean durations of openings and bursts at both Aβ concentrations were slightly briefer than the control but the differences were not statistically significant (Table 2). Due to the very brief durations, differences on these values may be inaccurate since they approach the resolution limit of our system. We therefore tested the effect of Aβ (10–1,000 nM) on ACh-activated channels in the presence 1 μM PNU-120596, which by increasing the duration of the activation episodes allows a better description of the molecular effects.

**Table 2:** Effect of Aβ on ACh-elicited channel activity.

PAM	Aβ	Aβ (nM)	*n*	Mean open duration (ms)	Mean cluster/burst duration (ms)
No	No	0	3	0.36 ± 0.07	0.77 ± 0.21
	Aβ_1–42_	10	3	0.26 ± 0.02	0.48 ± 0.06
		100	3	0.27 ± 0.04	0.49 ± 0.11
PNU-120596	No	0	6	159.6 ± 69.6	2263 ± 990
	Aβ_1–40_	50	3	176.7 ± 22.5	2220 ± 256
		100	5	61.6 ± 33.1*	698 ± 409*
		1,000	3	23.4 ± 22.0*	931 ± 303*
	Aβ_1–42_	10	5	61.1 ± 23.8*	888 ± 261*
		100	5	54.7 ± 47.8*	755 ± 560*
		1,000	6	59.6 ± 32.2*	883 ± 289*
NS-1738	No	0	12	4.2 ± 1.8	31.1 ± 8.8
	Aβ_1–40_	100	10	2.7 ± 1.1*	8.4 ± 4.8****
	Aβ_1–42_	100	4	2.1 ± 1.0*	10.7 ± 2.4****

Single-channel recordings from cells expressing human α7 were recorded in the presence of 100 μM ACh, 100 μM ACh plus 1 μM PNU-120596 or 100 μM ACh plus 10 μM N-(5-Chloro-2-hydroxyphenyl)-N′-[2-chloro-5-(trifluoromethyl) phenyl]urea (NS-1738). The effects of Aβ was evaluated for all conditions. The mean open and cluster durations were obtained from the corresponding histograms. Statistical comparisons were performed against the corresponding control condition using ANOVA (Dunnett’s multiple comparisons test). *p < 0.05, ****p < 0.0001.

**Figure 4:**
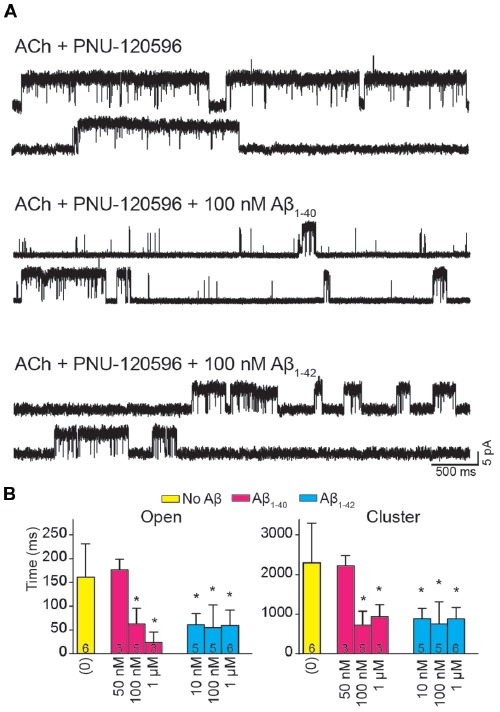
Aβ decreases the duration of activation episodes elicited by ACh and potentiated by PNU-120596. Single-channel recordings were performed in the cell-attached patch configuration at −70 mV membrane potential with 100 μM ACh and 1 μM PNU-120596 and in the absence or presence of Aβ. **(A)** Representative clusters are shown for each condition. Channels are shown as upward deflections. **(B)** Decrease of mean open and cluster durations by Aβ. Each value corresponds to the mean ± SD of different recordings. The number of recordings for each condition is shown in the bars. The cluster and open durations for each recording were obtained from the corresponding histograms and correspond to the slowest open components *p < 0.05.

The presence of Aβ_1–40_ or Aβ_1–42_ reduced the mean duration of openings and clusters elicited by ACh and potentiated by PNU-120596 as a function of concentration (Figure 4A). Whereas 50 nM Aβ_1–40_ did not affect open and cluster durations, 10 nM Aβ_1–42_ reduced these durations. The mean open and cluster durations were statistically significantly briefer than the control at concentrations equal and higher than 100 nM for Aβ_1–40_ and 10 nM for Aβ_1–42_ (*p* < 0.05, Table 2). At these concentrations, the mean durations were reduced about 3-fold (Figure 4B).

**Figure 5:**
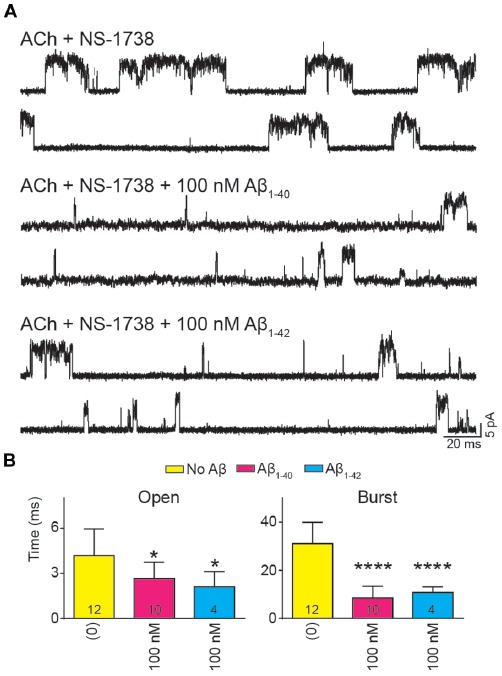
Aβ decreases the duration of events elicited by ACh and potentiated by the type I PAM N-(5-Chloro-2-hydroxyphenyl)-N′-[2-chloro-5-(trifluoromethyl) phenyl]urea (NS-1738). Single-channel recordings were performed in the cell-attached patch configuration at −70 mV membrane potential with100 μM ACh and 10 μM NS-1738 and in the absence or presence of Aβ. **(A)** Representative channel currents are shown for each condition. **(B)** Decrease of mean open and burst durations by Aβ. Each value corresponds to the mean ± SD of *n* different recordings (shown in the bars). The open and burst durations for each recording were obtained from the corresponding histograms and correspond to the slowest open components *p < 0.05, ****p < 0.0001.

To further determine if the effect of Aβ on α7 potentiation is specific for type II PAMs, we evaluated the action on channels activated by ACh and potentiated by NS-1738, which is a type I PAM (Timmermann et al., 2007; Andersen et al., 2016). In the presence of ACh, NS-1738 (10 μM) increased the mean open duration from about 0.3 ms to about 4 ms and opening events appeared grouped in bursts of about 30 ms (Figure 5, Table 2). In the presence of 100 nM Aβ_1–40_ or Aβ_1–42_, the mean burst duration was reduced to about 3-fold and the mean open duration was also statistically significantly briefer than in the control condition (Figure 5, Table 2).

Thus, we conclude that episodes elicited by ACh and potentiated by both types of PAMs are reduced by Aβ at high nanomolar concentrations.

## Discussion

Low concentrations (picomolar) of soluble Aβ peptides in the brain of healthy people have been reported to play physiological roles whereas in AD patients concentrations increase to the nanomole range and trigger the formation of insoluble plaques, a major neuropathologic hallmark of AD (Dineley, 2007; Parihar and Brewer, 2010; Collins-Praino et al., 2014; Puzzo et al., 2015). However, the possibility that Aβ oligomers play an important role in AD progression has gained weight (Miñano-Molina et al., 2011; Puzzo et al., 2015). Moreover, in the absence of plaques, intraneuronal accumulation of Aβ peptide has been shown to correlate with the initial steps in the tau-phosphorylation cascade, alterations in ERK2 signaling and impairment of higher CNS functions in rats (Echeverria et al., 2004a, b). Another feature of AD is the severe cholinergic deficit, which involves mainly α4β2 and α7 receptors. It has been established that α7 exhibits an exceptionally high Aβ affinity, an interaction that may influence synaptic transmission and plasticity and may also contribute to Aβ-mediated synaptic neural network dysfunction and to the severe cholinergic deficit (Wang et al., 2000; Buckingham et al., 2009; Puzzo et al., 2011; Dineley et al., 2015). Several reports have described the intracellular pathways involved in Aβ toxicity as well as crosstalk between Aβ- and α7-triggered signaling pathways (see review in Buckingham et al., 2009; Dougherty et al., 2003; Parri et al., 2011). However, the molecular mechanism by which Aβ affects α7 ionotropic activity is not well understood and has been explored mainly at the macroscopic current level. Thus, we took advantage of two different approaches to decipher the molecular basis of the direct actions of oligomeric Aβ at α7. Our spectroscopic results revealed that α7 adopts distinct stable conformations depending on the Aβ concentration range, and our single-channel recordings revealed that Aβ triggers α7 channel openings at low concentrations (picomolar to low nanomolar range) whereas at high concentrations (nanomolar to low micromolar range) it decreases the duration of ACh-elicited activation episodes. Both results fully support the idea that Aβ can act as an agonist and a negative modulator of α7 at different, physiologically attainable, concentrations.

One of the main concerns of working with Aβ is related to the standardization of the oligomeric preparations since aggregation is a dynamic and complex process, which is highly sensitive to preparation, experimental and analyzing conditions (Bitan et al., 2005; Buckingham et al., 2009; Hayden and Teplow, 2013; Watt et al., 2013). Thus, preparations are usually heterogenous since many types of soluble species co-exist. However, this scenario mimics the physiological/pathological situations where different oligomeric species and fibrils co-exist in a dynamic equilibrium (Walsh et al., 2000; Shankar et al., 2008; Noguchi et al., 2009; Santos et al., 2012; Esparza et al., 2016). Nevertheless, the results obtained with different Aβ oligomer preparations have yielded rather consistent results (Palop and Mucke, 2010). In particular, the effects exerted by our Aβ preparations, which are compatible with a heterogeneous array of oligomers and the absence of fibrils, are in general agreement with previous reported effects of oligomeric preparations on α7 (Liu et al., 2001; Dineley et al., 2002; Khan et al., 2010; Tong et al., 2011).

α7 ionotropic activity is characterized by very brief, sub-millisecond opening events and rapid desensitization (Bouzat et al., 2008; Corradi and Bouzat, 2016). We found that Aβ is capable of eliciting α7 activity either in the absence or presence of the type II PAM PNU-120596. In both conditions, activation by Aβ was favored at 100 pM or low nanomolar concentrations and was not detected at micromolar concentrations, in close agreement with macroscopic recordings of α7 expressed in oocytes (Dineley et al., 2002, 2015). Channel activity elicited by Aβ was significantly reduced with respect to that elicited by ACh in terms of the number of active patches and frequency of opening events. However, once opened, the mean open duration, cluster architecture and mean cluster duration were similar to those of ACh-elicited channel activity.

Conformational changes of α7 driven by the sole presence of Aβ at a low concentration were sensed by CrV. At 100 pM, oligomeric Aβ drove α7 conformation towards that of desensitized receptors but the subsequent addition of Carb allowed further conformational changes. Although this method senses conformations under equilibrium and cannot provide information about CrV K_D_ for open channels, the fact that in the presence of Aβ α7 is still responsive to Carb is compatible with the induction of both active and desensitized states. Nevertheless, the conformation of α7 in the presence of low Aβ and high Carb concentrations is different to that of the desensitized state (high Carb alone) as sensed by CrV, thus indicating the induction of a novel conformational state.

The enhancement of α7 activity as a protective role in AD and for the treatment of cognitive and memory impairment associated to neurological disorders appears to be well established (Inestrosa et al., 2013; Lendvai et al., 2013; Wallace and Bertrand, 2013; Uteshev, 2014; Dineley et al., 2015; Corradi and Bouzat, 2016; Yang et al., 2017). Also, a protective, physiological role has been proposed for soluble Aβ at low concentrations in healthy individuals (Giuffrida et al., 2009; Puzzo et al., 2015). Thus, the capability of low concentration Aβ preparations to activate α7 could be related to beneficial physiological effects. However, it would be expected that long-term exposure of an activator would lead to receptor desensitization.

Several reports have shown that high concentrations of Aβ have an inhibitory effect on the amplitude of α7-activated macroscopic currents as well as on signaling pathways (Dineley et al., 2002; Parri et al., 2011). In close agreement, we found that in the presence of PAMs the mean duration of activation episodes (clusters or bursts), which arise from a single receptor molecule, as well as the open channel lifetime are significantly reduced by high concentrations of oligomeric Aβ. There was also a trend of reduced mean durations in the absence of PAMs, but values were not statistically significantly different to the control. However, due to the very brief durations, which are close to the time-resolution limit of our system, such reduction may be underestimated. From a mechanistic point of view, the decreased duration of activation episodes and openings may be compatible with increased desensitization and/or channel block. We can discard fast open-channel blockade since brief closings corresponding to blocked openings (flickering) were not detected. However, enhanced desensitization and slow channel block processes are difficult to distinguish by electrophysiological techniques (Arias et al., 2009; Bouzat and Sine, 2018). CrV experiments showed that at high Aβ concentrations α7 adopts a conformational state which is not different to the resting state in terms of CrV K_D_ values and from this state α7 is not further reactive to Carb. In agreement with these observations, it was shown for the muscle nAChR that the channel blocker QX-314 does not change the CrV K_D_ value of the resting state and that this value remains constant even in the presence of Carb (Fabiani et al., 2018). Thus, we can infer that oligomeric Aβ at high concentrations behave as a slow channel blocker of α7. The reduced α7 activity in the presence of Aβ may contribute to the cholinergic signaling deficit and thus may be involved in the initiation and development of AD.

The combined action of PAMs and Aβ suggests that α7 potentiation by PAMs would be probably lower than expected in AD patients. By macroscopic current recordings it has been shown that Aβ_1–42_ inhibits α4β2 and α2β2 receptors and this inhibition is prevented in the presence of a PAM (Pandya and Yakel, 2011). These results are not in full disagreement with ours because, though reduced, we still detected potentiation. The characterization of the influence of Aβ on α7 potentiation contributes to a better extrapolation of the molecular effects of PAMs to their potential therapeutic effects.

A collateral but still important result of our study is the demonstration of different CrV binding profiles between α7 and muscle nAChRs. In the muscle nAChR, the CrV affinity for the desensitized state is greater than for the resting state whereas in α7 it is the other way around. CrV binds to luminal non-competitive antagonist sites which are localized in the channel vestibule (Lurtz and Pedersen, 1999). Our results showed that the conformation of this region is similar for both receptors in the resting state, but it is different in the desensitized state. Thus, the structural arrangements induced by prolonged exposure to Carb are different between muscle and α7 nAChRs. Overall, the use of a conformational probe has proved to be useful for revealing receptor subtype specific structural arrangements associated with functional states and opens doors to further studies in this respect.

The binding site of Aβ remains undefined. Computer docking studies suggested that Aβ may interact with α7 at agonist binding-site interfaces (Espinoza-Fonseca, 2004) and the conserved tyrosine 188 at Loop C of the agonist binding site was proposed to be involved in α7 activation by Aβ (Tong et al., 2011). On the other hand, the transmembrane cavity, a binding site of different allosteric ligands in the Cys-loop receptor family (Gill et al., 2011; Sauguet et al., 2015; Corradi and Bouzat, 2016), was proposed to be involved in the noncompetitive block of Aβ on α4β2 (Pandya and Yakel, 2011). Our spectroscopic results demonstrating that in the presence of low Aβ concentrations α7 is still reactive to Carb suggest that Aβ does not occupy orthosteric agonist binding sites and, in consequence, activation may be mediated by allosteric sites. Allosteric activation of α7 through a transmembrane site has been shown for 4BP-TQS. However, the activity profile is strikingly different to that of ACh, which is not the case of Aβ (Gill et al., 2011; Lasala et al., 2018). Alternatively, Aβ may not occupy the five orthosteric agonist sites but may still be able to induce activation; the remaining sites, subsequently occupied by Carb, may favor activation and desensitization. This possibility is in line with our previous reports showing that occupancy of only one of the five ACh-binding sites is required for α7 activation (Andersen et al., 2013). At high concentrations, Aβ may probably inhibit α7 by acting through an allosteric site, different from that of CrV since it does not interfere with its binding. Dual actions as low-efficacy agonists and channel blockers have been described for several compounds acting at different sites of nAChRs (reviewed in Bouzat and Mukhtasimova, 2018; Bouzat and Sine, 2018).

Overall, our study provides information from a molecular perspective to understand Aβ complex actions at the higher cellular level.

## Author Contributions

ML, CF, JC, SA and CB contributed to study design, analysis and interpretation of data. ML and CF: acquisition of data. CB and SA contributed to writing.

## Conflict of Interest Statement

The authors declare that the research was conducted in the absence of any commercial or financial relationships that could be construed as a potential conflict of interest.

## Abbreviations

ACh: acetylcholine
Carb: carbamylcholine
Aβ_1–42_: amyloid-β_1–42_ peptide
Aβ_1–40_: amyloid-β_1–40_ peptide
nAChR: nicotinic acetylcholine receptor
CrV: crystal violet
NS-1738: N-(5-Chloro-2-hydroxyphenyl)-N^′^-[2-chloro-5-(trifluoromethyl) phenyl]urea
PNU-120596: [N-(5-Chloro-2,4-dimethoxyphenyl)-N^′^-(5-methyl-3-isoxazolyl)-urea].
