# Supplementary material for: Molecular Modulation of Human α7 Nicotinic Receptor by Amyloid-β Peptides
Source: Front Cell Neurosci. 2019 Feb 8;13:37. doi: 10.3389/fncel.2019.00037 (PMC6376857; doi:10.3389/fncel.2019.00037)
Supplement: Supplementary file 1 [file Data_Sheet_1.PDF]

Supplementary Material

Molecular Modulation of Human α7 Nicotinic Receptor by Amyloid-β Peptides

Matías Lasala, Camila Fabiani, Jeremías Corradi, Silvia Antollini and Cecilia Bouzat*

***Correspondence:** Cecilia Bouzat, Instituto de Investigaciones Bioquímicas de Bahía Blanca (INIBIBB), Departamento de Biología, Bioquímica y Farmacia, Universidad Nacional del Sur (UNS)-Consejo Nacional de Investigaciones Científicas y Técnicas (CONICET), Camino La Carrindanga Km 7, Bahía Blanca, Argentina. Email: inbouzat@criba.edu.ar

Supplementary Figure 1

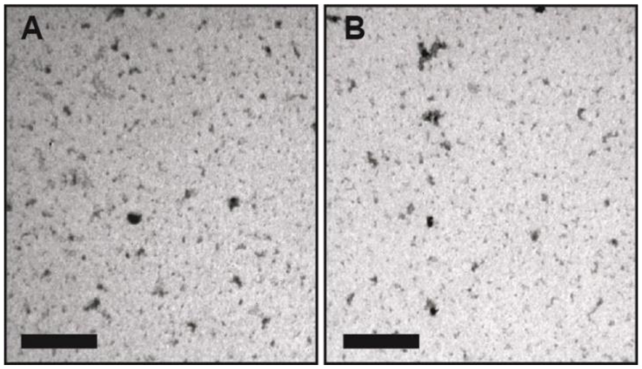

Supplementary Figure 1. Characterization of Aβ preparation. High-power (×100,000) electron photomicrograph showing Aβ preparations. Oligomeric $A\beta_{1\text{-}40}$ (A) or $A\beta_{1\text{-}42}$ (B) were prepared as described in Materials and Methods. Scale bar is 0.2 μm.
